# Estimating regional fossil fuel CO_2_ concentrations from ^14^CO_2_ observations: challenges and uncertainties

**DOI:** 10.1098/rsta.2022.0203

**Published:** 2023-11-27

**Authors:** Fabian Maier, Ingeborg Levin, Maksym Gachkivskyi, Christian Rödenbeck, Samuel Hammer

**Affiliations:** ^1^ Institut für Umweltphysik, Heidelberg University, INF 229,69120 Heidelberg, Germany; ^2^ ICOS Central Radiocarbon Laboratory, Heidelberg University, Berliner Strasse 53, 69120 Heidelberg, Germany; ^3^ Department Biogeochemical Systems, Max Planck Institute for Biogeochemistry, Hans-Knöll-Strasse 10, 07745 Jena, Germany

**Keywords:** fossil fuel CO_2_, radiocarbon, Δ^14^CO_2_ background, nuclear ^14^CO_2_ contamination

## Abstract

The direct way to estimate the regional fossil fuel CO_2_ surplus (ΔffCO_2_) at a station is by measuring the Δ^14^CO_2_ depletion compared with a respective background. However, this approach has several challenges, which are (i) the choice of an appropriate Δ^14^CO_2_ background, (ii) potential contaminations through nuclear ^14^CO_2_ emissions and (iii) masking of ΔffCO_2_ by ^14^C-enriched biosphere respiration. Here we evaluate these challenges and estimate potential biases and typical uncertainties of ^14^C-based ΔffCO_2_ estimates in Europe. We show that Mace Head (MHD), Ireland, is a representative background station for the Integrated Carbon Observation System (ICOS) atmosphere station network. The mean ΔffCO_2_ representativeness bias when using the MHD Δ^14^CO_2_ background for the whole observation network is of order 0.1 ± 0.3 ppm. At ICOS sites, the median nuclear contamination leads to 25% low-biased ΔffCO_2_ estimates if not corrected for. The ΔffCO_2_ masking due to ^14^C-enriched heterotrophic CO_2_ respiration can lead to similar ΔffCO_2_ biases as the nuclear contaminations, especially in summer. Our evaluation of all components contributing to the uncertainty of ΔffCO_2_ estimates reveals that, due to the small ffCO_2_ signals at ICOS stations, almost half of the ^14^C-based ΔffCO_2_ estimates from integrated samples have an uncertainty that is larger than 50%.

This article is part of the Theo Murphy meeting issue 'Radiocarbon in the Anthropocene'.

## 1. Introduction

Large uncertainties still exist in greenhouse gases budgets to fully understand the causes of their recent atmospheric changes [1]. The atmospheric boundary layer is the natural integrator of ground-level emissions. In conjunction with atmospheric transport models, trace gas observations in the boundary layer can, therefore, be used to estimate emissions or uptake rates of these gases (e.g. [2,3]). Including process-based emission models and inventories further helps to disentangle natural, e.g. climate-driven changes from anthropogenic emissions. However, there still exist large uncertainties in source attribution. Therefore, supplementary measurements, such as isotopic observations, have been added to the monitoring programmes to help distinguish different sources or constrain sink processes. In the case of CO_2_, the radioactive isotope radiocarbon (^14^C) has a prominent role, e.g. in separating fossil (radiocarbon-free) CO_2_ emissions from natural carbon fluxes between atmosphere, ocean and continental biosphere.

^14^C is a particularly useful tracer on regional and continental scales, where anthropogenic emissions from the burning of fossil fuels play an important role in the carbon budget [4–11]. Disentangling the influence of fossil emissions from ecosystem fluxes on the observed CO_2_ concentrations over densely populated areas would, on one hand, provide a means to follow the effectiveness of fossil CO_2_ emission reduction strategies (e.g. [12]), on the other hand, allows observing, e.g. climate-driven variations and long-term changes in ecosystem functioning directly from the atmosphere [10]. Co-located observations of CO_2_ and Δ^14^CO_2_ (for a definition of the Δ^14^CO_2_-notation that is generally used to report atmospheric ^14^CO_2_ observations, see §2a) over continents and in polluted areas can be exploited in two ways. One possibility is to derive ΔffCO_2_ estimates from the atmospheric Δ^14^CO_2_ differences between a background site and the (polluted) monitoring station in what we call here the regional isotope budget approach. Alternatively, the CO_2_ and Δ^14^CO_2_ observations can be used in a dual-tracer atmospheric inverse modelling framework to estimate the ffCO_2_ emissions directly [8,10].

The regional isotope budget approach is frequently used to estimate the share of the so-called recently added CO_2_ from fossil fuel burning and cement production (e.g. [5,6,13–17]). These studies determined the regional fossil CO_2_ excess at an observational station with respect to a non-polluted reference site. This reference or background site could be a high mountain station, which is sampling air from the free troposphere, or a marine site located at the boundary of a continent. The regional isotope budget approach provides the fossil CO_2_ excess concentration (ΔffCO_2_, which for clarity we will name C_ff_ in all equations and figures) directly, without the need to model the complete CO_2_ and ^14^CO_2_ cycles. For sites with a long-term Δ^14^CO_2_ record, this approach can also be used to investigate ffCO_2_ emission trends under the assumption that the atmospheric transport had no significant trend during the considered time period [12].

Typically, however, the ΔffCO_2_ estimates at an observation station show large variations not only on long but also on sub-seasonal time scales, which are mainly driven by atmospheric transport and mixing processes. If this variability can be represented by atmospheric transport models, the ΔffCO_2_ observations can be used in inverse modelling frameworks to estimate the ffCO_2_ emissions in the footprint of the station [18]. By contrast, the dual-tracer inversion framework [8,10,11] allows for direct usage of the CO_2_ and Δ^14^CO_2_ observations to estimate the ffCO_2_ emissions. But in this alternative approach *a priori* information is needed to represent the CO_2_ fluxes from the ocean and the terrestrial biosphere as well as their respective isotopic ^14^CO_2_ signatures. Furthermore, a representation of the atmospheric Δ^13^CO_2_ signature is required and the cosmogenic production of ^14^CO_2_ in the upper atmosphere and its (seasonal) transport into the troposphere must be implemented correctly.

Both, the regional isotope budget, and the dual-tracer inversion approach have, thus, advantages and disadvantages. While the dual-tracer inversion requires an *a priori* representation of the CO_2_ and ^14^CO_2_ fluxes and the related atmospheric gradients to directly estimate ffCO_2_ emissions, the regional isotope budget approach effectively assumes that these can be adequately captured by the appropriate selection of a representative background. Still, both approaches are not always straightforward to apply. In populated areas, we need to consider ^14^CO_2_ emissions, e.g. from nuclear installations, that contaminate the Δ^14^CO_2_ observations if such emitters are located in the footprints of the sites. Also, CO_2_ respired from decomposing organic material, e.g. in soils can mask part of the fossil signal. The biosphere had incorporated bomb ^14^C in the decades following the atmospheric nuclear bomb testing during the last century, and this ^14^CO_2_ is today released by heterotrophic respiration [19–21]. While these contaminating ^14^CO_2_ fluxes must be implemented in the dual-tracer inversion, the regional isotope budget approach uses estimates of correction terms to adjust the observed Δ^14^CO_2_ gradients regarding these masking effects. These correction terms are either based on expert judgement or estimated by simulating the Δ^14^CO_2_ contaminations with *a priori* fluxes and transport models.

Over the years, different assumptions have been made when applying the regional isotope budget approach to, e.g. account for the masking effects mentioned above. The aim of the present study is to in-depth re-visit the regional isotope budget approach, its underlying assumptions and investigate potential biases introduced in the respective results. We make sensitivity analyses to estimate the error contributions from all relevant components in this approach, including effects related to the choice of the background station. To obtain typical estimates of these potential biases and errors, we use the highly populated European continent as target region as this area has established a dense network of Δ^14^CO_2_ observations to validate fossil CO_2_ emissions and follow its potential changes [22,23].

## 2. Methods

### (a) The regional isotope budget approach to calculate recently added ffCO_2_

In the regional isotope budget approach, the CO_2_ concentration C_meas_ observed at a measurement station can be written as the sum of the background CO_2_ concentration C_bg_ and the recently added or removed CO_2_ contributions from different sources and sinks (e.g. [5,6]):

2.1 $${\text{C}}_{\text{meas}}={\text{C}}_{\text{bg}}+{\text{C}}_{\text{ff}}+{\text{C}}_{\text{resp}}+{\text{C}}_{\text{photo}}\;(+{\text{C}}_{\text{ocean}}+{\text{C}}_{\text{strato}}).$$

The contribution from the background C_bg_ accounts for the by far largest share and is globally increasing due to global anthropogenic emissions and land use change. The regionally added or removed CO_2_ contributions originate from the combustion of fossil fuels and cement production (C_ff_), the biosphere respiration (C_resp_) and photosynthesis uptake (C_photo_) in the target area. It may also contain a component from coastal ocean areas (C_ocean_) and may be influenced by air mass intrusions from the stratosphere (C_strato_). Each CO_2_ component in equation (2.1) is associated with a characteristic ^14^CO_2_ signature. When expressing these ^14^CO_2_ signatures in the so-called Δ-notation, i.e. the relative deviation of the ^14^C/C isotopic ratios from a standard material in permil (as introduced by Stuiver & Pollach [24]), the Δ^14^C signatures of the different components are directly comparable, since the Δ-notation accounts for mass-dependent isotopic fractionation during the different exchange processes between the corresponding reservoirs and the atmosphere and also corrects for radioactive decay between sampling and analysis times. Note that, in the following, we replace Δ^14^C by Δ^14^ to improve the readability of the equations below.

Since the sum over the products of the individual CO_2_ contributions and the associated isotopic signatures $\sum_{\text{i}}{\text{C}}_{\text{i}}\cdot \Delta_{\text{i}}^{14}$ is a conserved quantity [25], it follows that (see [6]):

2.2 $$\begin{array}{rl} {\text{C}}_{\text{meas}}\cdot \Delta_{\text{meas}}^{14} & \;={\text{C}}_{\text{bg}}\cdot \Delta_{\text{bg}}^{\text{14}}+{\text{C}}_{\text{ff}}\cdot \Delta_{\text{ff}}^{\text{14}}+{\text{C}}_{\text{resp}}\cdot \Delta_{\text{resp}}^{14}\\ & \;\;+{\text{C}}_{\text{photo}}\cdot \Delta_{\text{photo}}^{14}(+{\text{C}}_{\text{ocean}}\cdot \Delta_{\text{ocean}}^{14}+{\text{C}}_{\text{strato}}\cdot \Delta_{\text{strato}}^{14})\\ & \;\;+{\text{C}}_{\text{meas}}\cdot \Delta_{\text{nuc}}^{14}. \end{array}$$

The last term in equation (2.2) considers the potential contamination of the $\Delta_{\text{meas}}^{14}$ signature at the measurement site by ^14^CO_2_ emissions from nuclear facilities. These pure ^14^CO_2_ emissions cause a change in the measured Δ^14^CO_2_ ($\Delta_{\text{nuc}}^{14}$) at the station but no change in the observed CO_2_ concentration. For European stations the Integrated Carbon Observation System (ICOS) Carbon Portal (CP) provides a dedicated Jupyter notebook to calculate the contamination from nuclear facilities in Europe (freely available after registration at: https://www.icos-cp.eu/data-services/tools/jupyter-notebook, last access: 5 October 2022). With this Jupyter notebook the 3-hourly footprints from each ICOS station can be mapped with the annual mean ^14^CO_2_ emissions of the European nuclear facilities taken from the Radioactive Discharges Database (RADD). Hence, we use this Jupyter notebook to model for each Δ^14^CO_2_ sample an individual $\Delta_{\text{nuc}}^{14}$ contribution. The used footprints were calculated with the Stochastic Time-Inverted Lagrangian Transport (STILT, [26]) model. Note that the influence from these (elevated) point sources requires an additional modelling effort if the nuclear installation is located at a distance smaller than *ca* 50 km from the measurement site as shown by Maier *et al*. [27]. This modification has, however, not yet been implemented in the ICOS CP tool. We assume for the modelled $\Delta_{\text{nuc}}^{14}$ contributions an uncertainty of 100% to account for possible time-dependent (sub-annual) variations in the nuclear ^14^CO_2_ emissions as well as transport uncertainties. A brief description of how the nuclear ^14^CO_2_ contributions $\Delta_{\text{nuc}}^{14}$ are calculated and a more detailed explanation of their assumed uncertainty is given in appendix A.1. The regional isotope budget approach assumes that the background station contains all nuclear contributions from outside the target area and that contaminations from nuclear facilities inside the target area are negligibly small at the background station. This assumption should be appropriate if we use a background site at which only marine air is sampled.

In the further evaluations, we neglect the contributions from (i) the ocean and (ii) the stratosphere, because of the following reasons: (i) In Europe we mainly focus on continental measurement stations without direct oceanic influence. Therefore, we assume that the oceanic CO_2_ contribution and its Δ^14^ signature are integrated in the background measurements. This assumption should be appropriate if we use a marine background station; and (ii) we further assume that the latitudinal and vertical differences between the measurement site and the background station are small enough, so that both stations are similarly influenced by ^14^C-enriched stratospheric air; therefore, we assumed that also the stratospheric component is already integrated in the background measurements. Note that longitudinal differences in the stratospheric ^14^C production and corresponding influence on tropospheric Δ^14^C can be neglected (e.g. [28]).

As the ^14^C/C ratio of fossil CO_2_ is zero, the $\Delta_{\text{ff}}^{14}$ signature of fossil fuel combustion CO_2_ is −1000‰. Therefore, equation (2.2) can directly be used to calculate the regionally added ffCO_2_ contribution C_ff_ if all other CO_2_ contributions and Δ^14^ signatures are known. However, as the two biospheric CO_2_ components, C_photo_ and C_resp_, are typically not known separately, it is convenient to use equation (2.1) for eliminating the (potentially poorest known) CO_2_ contribution from photosynthesis C_photo_ in equation (2.2). As the Δ-notation accounts for mass-dependent isotopic fractionation, we can directly use the Δ^14^ signature of the photosynthesized atmospheric CO_2_ to substitute the $\Delta_{\text{photo}}^{14}$ in equation (2.2). However, the Δ^14^ signature of an air mass changes on its path from the background station to the measurement site and therewith also the Δ^14^ signature taken up by photosynthesis $\left(\Delta_{\text{photo}}^{14}\right)$. Previous studies have either approximated the $\Delta_{\text{photo}}^{14}$ signature of the photosynthetically absorbed CO_2_ with $\Delta_{\text{bg}}^{14}$ or with $\Delta_{\text{meas}}^{14}$. If $\Delta_{\text{photo}}^{14}$ is approximated by the $\Delta_{\text{meas}}^{14}$ value of the ambient CO_2_, the ffCO_2_ excess can be calculated according to:

2.3 $${\text{C}}_{\text{ff}}={\text{C}}_{\text{bg}}\cdot \frac{\Delta_{\text{bg}}^{14}-\Delta_{\text{meas}}^{14}}{\Delta_{\text{meas}}^{14}+1000\text{‰}}+{\text{C}}_{\text{meas}}\cdot \frac{\Delta_{\text{nuc}}^{14}}{\Delta_{\text{meas}}^{14}+1000\text{‰}}+{\text{C}}_{\text{resp}}\cdot \frac{\Delta_{\text{resp}}^{14}-\Delta_{\text{meas}}^{14}}{\Delta_{\text{meas}}^{14}+1000\text{‰}}.$$

Otherwise, if $\Delta_{\text{photo}}^{14}$ is approximated with the background $\Delta_{\text{bg}}^{14}$ signature, one gets:

2.4 $${\text{C}}_{\text{ff}}={\text{C}}_{\text{meas}}\cdot \frac{\Delta_{\text{bg}}^{14}-\Delta_{\text{meas}}^{14}}{\Delta_{\text{bg}}^{14}+1000\text{‰}}+{\text{C}}_{\text{meas}}\cdot \frac{\Delta_{\text{nuc}}^{14}}{\Delta_{\text{bg}}^{14}+1000\text{‰}}+{\text{C}}_{\text{resp}}\cdot \frac{\Delta_{\text{resp}}^{14}-\Delta_{\text{bg}}^{14}}{\Delta_{\text{bg}}^{14}+1000\text{‰}}.$$

Which of the approximations, equation (2.3) or (2.4), better estimates regional ΔffCO_2_ depends on how representative the respective $\Delta_{\text{photo}}^{14}$ approximation is for the biosphere affecting the measurement site. Equation (2.3), for example, is suitable for remote stations that are significantly influenced by the local biosphere. Therefore, the $\Delta_{\text{photo}}^{14}$ signature of the (mainly contributing) local biosphere is best approximated by the measured $\Delta_{\text{meas}}^{14}$ signature of the ambient air CO_2_ at the measurement site. By contrast, equation (2.4) might be applicable for stations with very little influence of the local biosphere and supposedly large local fossil emissions. Thus, it might be less appropriate to approximate the $\Delta_{\text{photo}}^{14}$ signature of the photosynthetically absorbed air with the strongly depleted ambient air $\Delta_{\text{meas}}^{14}$ signature. Indeed, for such cases the $\Delta_{\text{bg}}^{14}$ signature might more accurately describe the $\Delta_{\text{photo}}^{14}$ signature of the (mainly contributing) far field biosphere. The difference between using the two alternative equations will be evaluated in §3a(i).

### (b) Importance of the components and correction terms in the regional isotope budget approach for estimating ΔffCO_2_

The choice of the background station is crucial for the ΔffCO_2_ estimate, since the first term in equations (2.3) and (2.4) is proportional to the Δ^14^ difference between the measurement station and the background station and usually contributes the most. Ideally, a single site can be used to provide a physically representative background for all other observation sites within a Δ^14^CO_2_ monitoring network. This will be the case when all observation sites within the sampling domain are influenced by the same weather systems, generally flowing from the background to the observation sites. In the modelling world, this would mean that the unique background should be valid for all boundaries of the targeted domain, for which the ffCO_2_ flux shall be estimated. This assumption of representativeness is examined further in §3a(ii).

The second term in equations (2.3) and (2.4) describes the corrections for nuclear contaminations at the measurement site. Particularly in Europe with many nuclear power plants and two large nuclear fuel reprocessing plants (cf. figure 8), disregarding these nuclear contaminations would result in significant underestimation of ΔffCO_2_ estimates [5,29,30].

The third term in equations (2.3) and (2.4) accounts for the correction for biosphere respiration, which, if not accounted for, may also mask part of the ΔffCO_2_. The $\Delta_{\text{resp}}^{14}$ signature differs for autotrophic and heterotrophic respiration. In earlier studies (e.g. [13]), we approximated the autotrophic $\Delta_{\text{resp}}^{14}$ signature with that of background air, while for the heterotrophic component, we used the Δ^14^C signature modelled by Naegler & Levin [31]. Turnbull *et al*. [6] assumed a mean terrestrial carbon residence time of 10 ± 10 years and used the ^14^C history of the Northern Hemisphere to calculate the Δ^14^ signature of heterotrophic respiration. $\Delta_{\text{soil}}^{14}$ of soil respiration was measured in summer 2012 at a boreal forest site in Finland by Palonen *et al*. [32] to lie between (48.2–56.7)‰, values about 20‰ higher than background air in that year. Chanca [33] reported a mean $\Delta_{\text{resp}}^{14}$ of 32.0 ± 7.4‰ for a tropical rainforest site in Brazil. This latter value was on average 29‰ higher than atmospheric CO_2_ in that year. From these studies, we conclude that $\Delta_{\text{resp}}^{14}$ in the last decade was a few tens of ‰ higher than the contemporary atmospheric CO_2_. We used the Vegetation Photosynthesis and Respiration Model (VPRM, [34]) in combination with STILT to simulate the respiration CO_2_ signal C_resp_ for the two ICOS sites Observatoire Pérenne de l'Environnement (OPE) in France and Křešín (KRE) in the Czech Republic. These results show on average about twice as high C_resp_ signals in summer than in winter. Overall, we would expect the typical range of C_resp_ to be between 2 and 8 ppm at ICOS sites in Central Europe.

## 3. Results

The aim of the present study is to quantify potential biases and estimate individual error contributions to the total uncertainty of ΔffCO_2_ resulting from the uncertainties of the measured and estimated parameters and corrections in equations (2.3) and (2.4). In §3a, we first evaluate the difference of results when approximating $\Delta_{\text{photo}}^{14}$ by $\Delta_{\text{meas}}^{14}$ or $\Delta_{\text{bg}}^{14}$, respectively. Then, as a typical example for a populated region, we evaluate biases introduced in the ΔffCO_2_ result when selecting only a single background station for Europe that is located on the western coast of Ireland. We will further quantify the biases in the ΔffCO_2_ estimates, which would be induced when ignoring nuclear contaminations, and finally, we investigate the bias related to Δ^14^C-enriched respiration CO_2_. Section 3b investigates the relative uncertainty contributions introduced through (i) the uncertainty of the CO_2_ and Δ^14^CO_2_ observations at the measurement station, (ii) the uncertainty of the background Δ^14^CO_2_ curve, which incorporates the uncertainty of its construction (smooth curve fitted through observational data at that background station) as well as the uncertainty due to the representativeness of the background curve. We will (iii) estimate the uncertainty of the correction for nuclear ^14^CO_2_ contamination and (iv) the error contribution of the correction for heterotrophic respiration. Finally, we assess the total uncertainty of typical (bias-corrected) ΔffCO_2_ estimates at European ICOS stations by identifying those components/parameters with the largest impact.

### (a) Potential bias components in the ΔffCO_2_ estimates

#### (i) Bias due to the approximation of $\Delta_{\text{photo}}^{14}$ (choice of equation (2.3) or (2.4))

**Figure 1.**
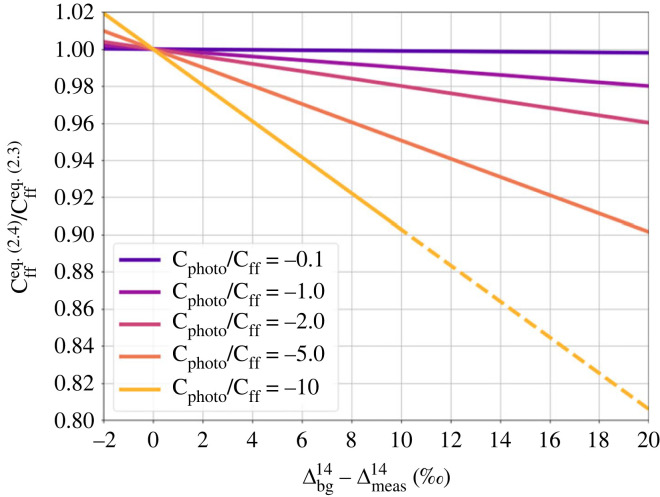
Ratio of ΔffCO_2_ when estimated with equation (2.4) versus equation (2.3) for C_photo_/C_ff_ ratios ranging from −0.1 to −10.

To investigate the impact of the approximation of $\Delta_{\text{photo}}^{14}$ on the ΔffCO_2_ estimates, i.e. the difference between equation (2.3) and (2.4), figure 1 shows the ratio of the ΔffCO_2_ results from equation (2.4) to (2.3) plotted versus the $\left(\Delta_{\text{bg}}^{\text{14}}-\Delta_{\text{meas}}^{14}\right)$ difference for typical current Δ^14^CO_2_ and CO_2_ values (cf. table 1) and a large range of possible ratios of C_photo_ to C_ff_. As can be seen from figure 1, the differences in the final ΔffCO_2_ results when using equation (2.4) versus (2.3) can be large at stations where the (negative) photosynthetic component is much larger than the (positive) ΔffCO_2_ component and if the Δ^14^CO_2_ difference between station and background is large. Typical $\left(\Delta_{\text{bg}}^{14}-\Delta_{\text{meas}}^{14}\right)$ differences at ICOS stations go up to 10‰ (cf. figure 6 below), and in cases with high differences (large ΔffCO_2_), i.e. in winter, the photosynthetic uptake is generally small, so that we expect |C_photo_/C_ff_| ratios < 2 (figure 9). Therefore, we expect biases between using equation (2.3) relative to equation (2.4) to be restricted to a few per cent only. In summer, the |C_photo_/C_ff_| ratio is larger but the Δ^14^CO_2_ difference to the background tends to be smaller, again pushing the difference between the equations into the range of few per cent. As we are evaluating here typical biases and uncertainties of ^14^C-based ΔffCO_2_ at ICOS stations that are typically located more than 40 km away from large ffCO_2_ emitting regions [35], the photosynthetic uptake signals will most probably be larger than those from fossil emissions (figure 9). Therefore, we restrict our further analysis on ΔffCO_2_ estimates applying equation (2.3).

**Table 1.** Assumed values and parameters for our standard uncertainty evaluation.

parameter	value ± uncertainty	description
${\text{C}}_{\text{meas}}$	(420 ± 0.1) ppm	CO_2_ concentration at station
$\Delta_{\text{meas}}^{14}$	±2‰	typical Δ^14^CO_2_ measurement uncertainty
C_bg_	(410 ± 1) ppm	background CO_2_ concentration
$\Delta_{\text{bg}}^{14}$	(3 ± 1)‰	taken from fitted background curve of MHD data
$\Delta_{\text{nuc}}^{14}$	(1 ± 1)‰	typical nuclear Δ^14^CO_2_ contamination at ICOS stations
${\text{C}}_{\text{resp}}/{\text{C}}_{\text{ff}}$	3 ± 3	respiration CO_2_ component relative to ΔffCO_2_
$\Delta_{\text{resp}}^{14}$	(25 ± 12)‰	respiration Δ^14^CO_2_ signature

#### (ii) Biases due to the choice of one background station

**Figure 2.**
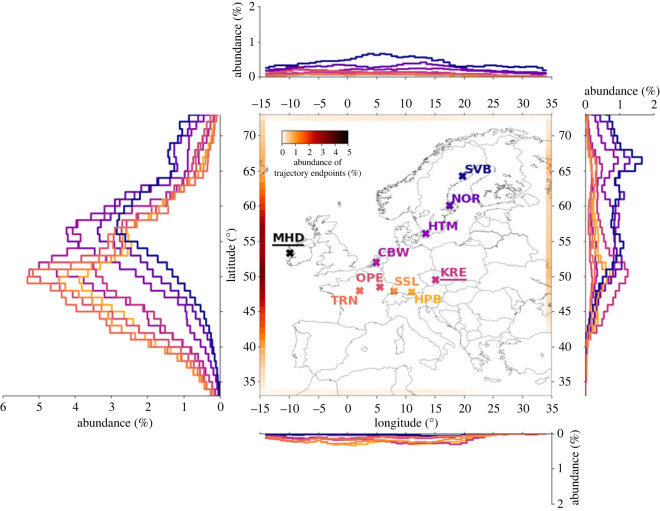
Abundance distribution for the endpoints of the STILT back-trajectories from nine ICOS sites (coloured crosses) in 2018. At each hour and each station, 100 particles were released in STILT, and their back-trajectories were calculated for 10 days backward in time. If the trajectory leaves the shown domain, its endpoint is defined as the grid cell where the trajectory leaves the domain the first time. STILT was driven with the 0.25° resolved ERA5 (European ReAnalysis 5) meteorology from the European Center for Medium-Range Weather Forecasts (ECMWF). The black cross indicates the position of the Mace Head (MHD) background site (back-trajectories were not calculated for MHD).

*Representation of background air with respect to individual ICOS stations:* The regional isotope budget approach estimates ΔffCO_2_ with respect to a (measured) background. It implicitly assumes that the air masses arriving at the stations started with this background value. In order to use the ΔffCO_2_ estimates to obtain information on fossil emissions in a given target area, it is necessary to assess the implicit assumption that the selected background is representative of all boundaries of this target area. We examine the representativeness problem using Europe and the ICOS observation network as an example target area. We base our evaluation on the standard STILT domain over Europe, which extends from 33° N to 73° N and from 15° W to 35° E (figure 2). The distribution of the typical air mass origins entering the STILT domain is constructed by the endpoints of hourly 10-days STILT back-trajectories for the year 2018 for nine ICOS stations, where regular Δ^14^CO_2_ observations are conducted. The abundance distributions of those trajectory endpoints at the four boundaries (figure 2) clearly indicate that these central European stations are predominantly influenced by westerly winds, which transport Atlantic air masses to the European continent. On average, 67% of the back-trajectories from the ICOS sites end at (or go beyond) the western boundary of the model domain, with an accumulation between roughly 45° and 60° N. This is a good argument to select Mace Head (MHD, 53.33° N, 9.90° W, 5 m a.s.l.), located close to the western boundary of the STILT domain, as a background site to calculate the ffCO_2_ excess at European (ICOS) stations. MHD is located on the west coast of Ireland and atmospheric ^14^CO_2_ samples are only collected during situations when the air comes from the marine sector. These Δ^14^CO_2_ (together with CO_2_) background measurements can thus be assumed as representative for the Atlantic boundary of the European continent. However, they may be less suitable as background reference in situations when the measurement stations are influenced by easterly (or southerly) air masses, which transport potentially polluted continental air to our domain. Also northerly Arctic air masses may have a different Δ^14^CO_2_ level. Figure 2 shows that for on average 33% of the time in 2018 the air masses originated from non-western boundaries (or from within the target area) with on average 13% of all situations in 2018 entering via the eastern boundary.

**Figure 3.**
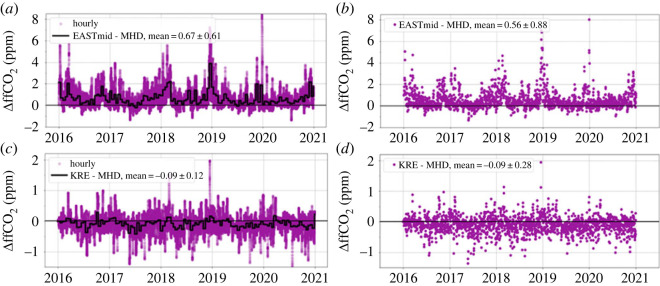
TM3-simulated fossil fuel CO_2_ concentration offsets relative to Mace Head caused by emissions outside of our target region (figure 2). (*a*,*b*) The offsets at a location at mid-latitudes of the eastern boundary, for all hours (*a*) and for 13 h UTC only (*b*). (*c*,*d*) Corresponding data for the ICOS station Křešín (KRE). Note the different scales in (*a*,*b*) and (*c*,*d*).

*Background variability at boundaries of the target area:* Currently, no background Δ^14^CO_2_ observations exist in eastern Europe or close to the eastern boundary of our target domain. This makes it impossible to construct a purely observation-based Δ^14^CO_2_ reference for the eastern air masses entering our target region and to deduce the bias of the representativeness problem from this. Therefore, we used the global atmospheric tracer transport model TM3 ([36]; spatial resolution of 4° × 5°) to estimate ffCO_2_ concentrations of air masses entering the eastern boundary of our domain. For this simulation, ffCO_2_ emissions within the European target domain have been set to zero, while we used the Global Carbon Budget Gridded Fossil Emissions Dataset (GCP-GridFED; v.2022.2) emissions [37] elsewhere. Figure 3*a*,*b* shows the simulated ffCO_2_ concentration difference of hourly data (purple dots) between a virtual station located at the eastern boundary of the STILT domain at mid-latitudes (55° N, 34° E, 150 m a.g.l.) and MHD for the years 2016–2020. As expected, this difference is generally positive. It varies between a few tenths of a ppm up to more than 8 ppm in one event in 2020. A significant seasonal variation is observed, with higher concentration differences compared with MHD during winter and lower differences in summer. This seasonality is mainly due to seasonal variations of emissions, but also due to seasonal variations in atmospheric transport. As emissions within the target domain were set to zero in this TM3 model run, the concentration differences in figure 3*a*,*b* provide a good approximation of the ffCO_2_ concentration offset (relative to MHD) of air masses entering the domain from the east. Figure 3*a* shows the biases for all hours in 2016–2020 and for averages of two-week integrated samples (black solid line). Figure 3*b* shows the difference for potential flask samples collected at midday. For any potential station located close to this border, this difference would represent the approximate positive bias of the calculated ΔffCO_2_ for those situations when the station is *not* influenced by westerly air masses but by air from the east. The mean bias is about 20% larger for all hours and two-week integrated samples than for flask samples collected at midday. For flask samples, however, the standard deviation of that bias is about 30% higher than for two-week integrated samples since flask samples depend stronger on individual weather situations. The station in the ICOS network located closest to the eastern border of the target domain is Křešín (KRE). This station typically experiences about 16% of situations with easterly trajectories (figure 2). On average over *all* situations of potential flasks sampled at Křešín at midday, the ΔffCO_2_ bias would thus be only 0.56 × 0.16 = 0.09 ppm. However, Křešín station is located about 20° west of the eastern boundary. Therefore, any contribution from domain-external ffCO_2_ emissions coming from the east will be diluted during transport of the air mass to the station.

We can use similar arguments and the TM3 model results to estimate the influence on ICOS stations due to emissions from outside our domain from the west, e.g. from North America. The concentration from such sources as observed at MHD would also be (further) diluted when the air mass travels over Europe and reaches, e.g. the Křešín station. This is why in figure 3*c*,*d*, where we plot the difference of the TM3 concentrations simulated for KRE minus those at MHD, often turn out to be negative. Note that the positive values in this plot are the signals from the easterly trajectories, but here diluted during the transport from the eastern boundary to the Křešín site. As the majority of trajectories reaching Křešín comes from the west, the average difference is negative. In summary, we expect typical mean representativeness biases of order (0.1 ± 0.3) ppm ΔffCO_2_ when applying the regional isotope budget approach for the investigated example of the ICOS station network with MHD as a background in the STILT domain. A ffCO_2_ inversion using our ΔffCO_2_ estimates would incorrectly locate this (0.1 ± 0.3) ppm representativeness bias within the STILT domain.

*Influence of a latitudinal* Δ^14^CO_2_ *gradient:* The MHD background station is located at about 55° N, a latitude where global fossil CO_2_ emissions are large. We would, thus, expect lower fossil CO_2_ concentrations at lower latitudes of the Northern Hemisphere, corresponding to slightly higher Δ^14^CO_2_ in background air further south. Levin *et al*. [38] published mean Δ^14^CO_2_ data from Izaña station on Tenerife Island (28.3° N, 16.48° W, 2373 m a.s.l.) in the Atlantic Ocean, which indeed showed slightly higher values in the 1990s. However, in the last decade, the Δ^14^CO_2_ difference to MHD is smaller than 1‰. As air masses only occasionally arrive from latitudes south of 40° N, the potential bias due to a latitudinal Δ^14^CO_2_ gradient is assumed negligible and thus not taken into account here as a potential bias.

#### (iii) Biases due to nuclear ^14^CO_2_ emissions

**Figure 4.**
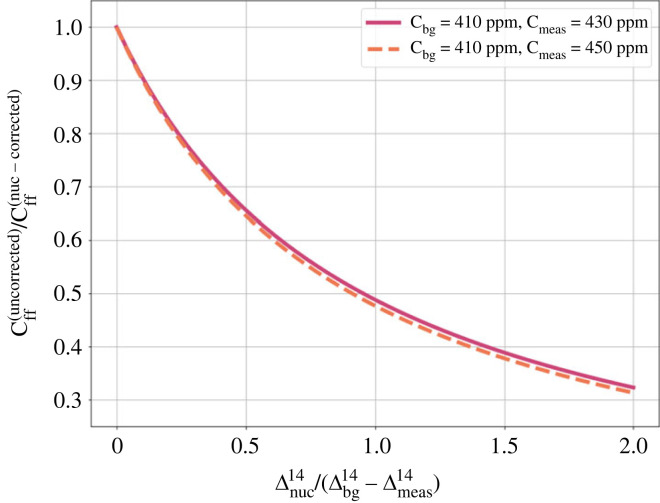
ΔffCO_2_ masking by ^14^CO_2_ emissions from nuclear installations in relation to the ratio between $\Delta_{\text{nuc}}^{14}$ and $\left(\Delta_{\text{bg}}^{14}-\Delta_{\text{meas}}^{14}\right)$.

As mentioned before, ^14^CO_2_ contaminations by emissions from nuclear installations are a potentially serious problem for ^14^C-based ΔffCO_2_ estimates in Europe ([29,30]; figure 8 for a distribution of the nuclear ^14^CO_2_ emissions in Europe). This may be particularly true at stations with small ΔffCO_2_ signals, i.e. small Δ^14^CO_2_ depletion at the measurement station compared with the background $\left(\Delta_{\text{bg}}^{14}-\Delta_{\text{meas}}^{14}\right)$. We, therefore, estimated the masking of ΔffCO_2_ $\left({\text{C}}_{\text{ff}}^{\text{uncorrected}}/{\text{C}}_{\text{ff}}^{\text{nuc - corrected}}\right)$ with respect to the ratio between $\Delta_{\text{nuc}}^{14}$ and $\left(\Delta_{\text{bg}}^{14}-\Delta_{\text{meas}}^{14}\right)$. As this masking effect (second term in equation (2.3)) also depends on the CO_2_ concentration at the measurement station, we estimated the masking for two typical concentrations, C_meas_ = 430 and 450 ppm, respectively, as displayed in figure 4.

A relative $\Delta_{\text{nuc}}^{14}$ contamination from nuclear emissions of about 25% of the $\left(\Delta_{\text{bg}}^{\text{14}}-\Delta_{\text{meas}}^{14}\right)$ signal would mask about 20% of the ΔffCO_2_ signal. The nuclear correction is, thus, especially important for stations with small Δ^14^C-depletions compared with the background site, as indeed expected at ICOS atmosphere stations (see below, figure 6). Sampling during times with potential influence from nuclear installations should, therefore, be avoided, e.g. for flask sampling, or the bias must be calculated precisely (see §3b(iv)).

#### (iv) Biases due to ^14^C-enriched biosphere respiration

**Figure 5.**
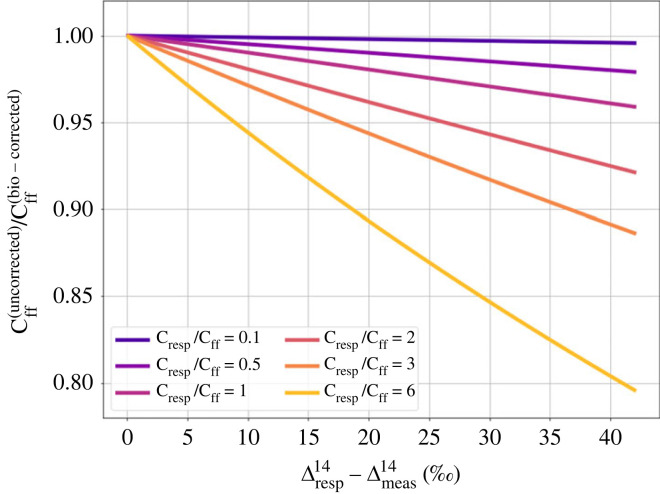
ΔffCO_2_ masking by ^14^C-enriched respiration CO_2_ plotted versus the difference between $\left(\Delta_{\text{resp}}^{14}-\Delta_{\text{meas}}^{14}\right)$ for C_resp_/C_ff_ ratios between 0.1 and 6.

Also soil respiration can mask part of the fossil fuel-related Δ^14^C difference between the stations and the background site. In this case, however, the contaminating source does not emit pure ^14^CO_2_, as is the case for nuclear installations. The respired CO_2_ today, if at all, is only slightly enriched in Δ^14^C compared with contemporary ambient CO_2_. Unfortunately, only very few measured data are available on $\Delta_{\text{resp}}^{14}$ in different ecosystems. These data suggest enrichments of a few tens of ‰ compared with atmospheric CO_2_ [32,33]. Figure 5 shows the masking of ΔffCO_2_ $\left({\text{C}}_{\text{ff}}^{\text{uncorrected}}/{\text{C}}_{\text{ff}}^{\text{bio - corrected}}\right)$ in relation to the Δ^14^ difference between respired and measured CO_2_ $\left(\Delta_{\text{resp}}^{14}-\Delta_{\text{meas}}^{14}\right)$ for a range of C_resp_/C_ff_ ratios between 0.1 and 6.

If the C_resp_ signal has a similar magnitude as ΔffCO_2_ (i.e. C_resp_/C_ff_ = 1), which is a typical ratio in winter (figure 9), the uncorrected ΔffCO_2_ is underestimated by less than 5%, under the assumption that the Δ^14^ difference between respiration CO_2_ and measured CO_2_ is smaller than 40‰. Apparently, this bias increases with an increasing relative C_resp_ signal. Thus, the biosphere correction is potentially important at stations with low ΔffCO_2_ and a high respiration component C_resp_, which is the case for a number of ICOS stations in summer and in Northern Europe, i.e. far away from high ffCO_2_ emission areas. Here we can find C_resp_/C_ff_ ratios as large as 6 (figure 9). In such situations, the ΔffCO_2_ masking due to ignoring the biosphere correction (third term in equation (2.3)) could become as large as 20%, if the Δ^14^ difference between respiration CO_2_ and ambient air CO_2_ is larger than 40‰.

#### (v) Typical Δ^14^CO_2_ signals at ICOS stations

**Figure 6.**
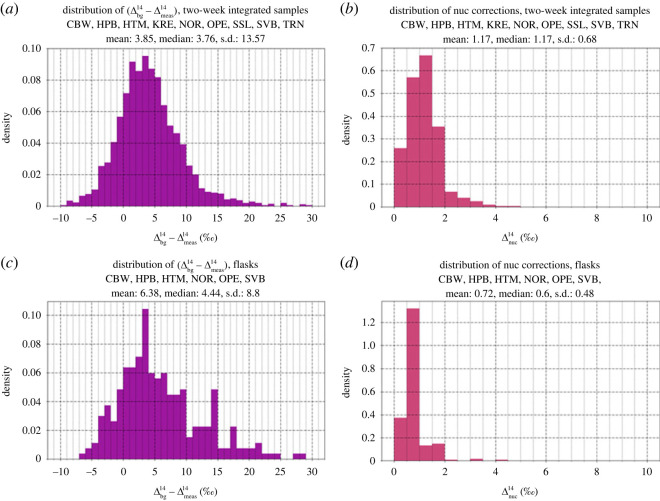
Distribution of observed Δ^14^ signals between reference station (MHD) and ICOS stations ((*a*) for two-week integrated samples and (*c*) for the currently available flask samples). (*b*,*d*) The distributions of the estimated nuclear corrections of the samples.

The relevance of the two bias correction terms for nuclear and respiration contamination at ICOS stations discussed above depends on their individual influence areas with respect to fossil emitters and ^14^CO_2_-emitting nuclear installations. It also depends on the type of samples collected (two-week integrated versus midday flasks). Figure 6*a* shows the distribution of $\left(\Delta_{\text{bg}}^{14}-\Delta_{\text{meas}}^{14}\right)$ of two-week integrated samples collected in the years 2017–2020 at the nine ICOS stations shown in figure 1. Figure 6*b* gives the distribution of the mean $\Delta_{\text{nuc}}^{14}$ contaminations for the same stations and sample type. The median measured Δ^14^CO_2_ difference between station and background (MHD) is only 3.8‰ while the median nuclear contamination amounts to about 30% of that value. If not corrected, this would correspond to an almost 25% masking of ΔffCO_2_. This illustrates the importance of the nuclear contamination problem in Europe and the need to correctly model $\Delta_{\text{nuc}}^{14}$. Such a correction could best be done with high-resolution emissions data and a reliable transport modelling system. Currently, however, only annual mean ^14^CO_2_ emission data are available.

Figure 6*c*,*d* shows similar distributions as *a*,*b*, but for hourly flask samples collected at 13 h local time. The median $\left(\Delta_{\text{bg}}^{14}-\Delta_{\text{meas}}^{14}\right)$ difference for the currently available flask samples—yet collected only at seven ICOS class-1 stations—is 4.4‰, slightly larger than for two-week integrated samples, while the nuclear correction term is, on average, only half of that for the integrated samples. Still the average masking of about 15% is significant and should be corrected.

### (b) Uncertainty of the ΔffCO_2_ estimates

After having quantified potential biases when using (i) equation (2.4) instead of equation (2.3) to estimate recently added ffCO_2_, (ii) the potential bias due to the use of a single background station for all European ICOS sites, (iii) neglecting corrections for nuclear ^14^CO_2_ emissions and (iv) for ΔffCO_2_ masking due to ^14^C-elevated biosphere respiration, in the following we evaluate the contributions to the total uncertainty of the bias-corrected ΔffCO_2_, including the uncertainty due to these bias corrections.

#### (i) Typical measurement uncertainty

One important uncertainty contribution to estimate recently added fossil CO_2_ is the Δ^14^CO_2_ measurement uncertainty. This uncertainty is relevant for the measurements at the ICOS sites but also for estimating the background reference, here from MHD data. Both determine the uncertainty of the main term in equations (2.3) and (2.4). Typical high-precision radiocarbon laboratories measure single atmospheric ^14^CO_2_ samples with a precision between Δ^14^C = (1.7–2.3)‰ (e.g. [39]). This range also covers the average precision and long-term repeatability in the ICOS Radiocarbon Laboratory for integrated and flask samples. For simplicity we assume here a measurement precision of 2‰ for all Δ^14^CO_2_ analyses.

#### (ii) Uncertainty of the ^14^CO_2_ background estimate

The uncertainty of the ^14^CO_2_ background estimate comprises two components. On the one hand, the uncertainties resulting from the construction of the ^14^CO_2_ background curve from the measurements at the background station, and on the other hand, the uncertainties resulting from the representativeness assumption. As illustrated in §3a(ii), using MHD as single background for all ICOS stations in western and central Europe could lead to a mean ΔffCO_2_ bias of order 0.1 ppm for two-week integrated samples and also for flask samples collected over one hour at midday at the most easterly located station KRE. The standard deviation of this bias was estimated to 0.12 ppm for integrated and to 0.28 ppm for flask samples, with individual biases of up to 2 ppm in exceptional cases. The variability of the bias can be seen as the representativeness uncertainty of the ^14^CO_2_ background.

To construct a continuous Δ^14^CO_2_ background curve for MHD that is applicable for flask and integrated samples, we calculated a smooth curve through these data by using a curve fitting routine developed by the National Oceanic and Atmospheric Administration (NOAA) [40]. A detailed description of how the background curve has been calculated is given in Appendix A.2. Two different uncertainty estimates have been made for the background reference, one based on the standard NOAA routine and a second one using a Monte Carlo approach. From these estimates we derived a mean uncertainty for $\Delta_{\text{bg}}^{14}$ of ±0.86‰. Adding the (independent) background representativeness uncertainty mentioned above, we obtain a total background reference uncertainty for integrated samples of 0.9‰ and for flask samples of 1.0‰. The uncertainty of the background CO_2_ reference concentration curve is negligible for the overall ΔffCO_2_ uncertainty, even if an assumed upper-limit CO_2_ background representativeness uncertainty of 5 ppm is added.

#### (iii) Uncertainty of the nuclear and respiration masking corrections

In order to estimate the error contributions of the two correction terms in equation (2.3) (masking by nuclear ^14^CO_2_ emissions, second term, and contribution from respiration of ^14^C-enriched CO_2_, third term), we made the following assumptions: we assume 100% uncertainty for the nuclear contamination estimates; this is justified with a significant transport model error to correctly simulate the dispersion and location of power plant plumes and by the fact that the significant temporal variability of the nuclear emissions (e.g. [30]) is totally ignored when using the currently reported annual mean emissions from RADD for estimating $\Delta_{\text{nuc}}^{14}$ (see appendix A.1). The ΔffCO_2_ masking contribution from CO_2_ respiration, C_resp_, has also a significant uncertainty because for daytime situations it has to be estimated using, e.g. a vegetation model that is coupled to an atmospheric transport model. For night-time situations when photosynthetic uptake of CO_2_ is negligible, C_resp_ or C_resp_/C_ff_, the latter being the relevant parameter that determines the masking (cf. figure 5), could be estimated in an iterative way as the difference between C_meas,_ C_bg_ and C_ff_. Finally, we estimate the uncertainty of $\Delta_{\text{resp}}^{14}$ to be 50% of the difference between $\Delta_{\text{resp}}^{14}$ and $\Delta_{\text{meas}}^{14}$. This means, if we measure a $\Delta_{\text{meas}}^{14}$ of 1‰ and assume a $\Delta_{\text{resp}}^{14}$ signature of 25‰, the applied uncertainty for $\Delta_{\text{resp}}^{14}$ would be 12‰. The dependence of the $\Delta_{\text{resp}}^{14}$ uncertainty on the $\left(\Delta_{\text{resp}}^{14}-\Delta_{\text{meas}}^{14}\right)$ difference seems appropriate as the biosphere correction (third term in equation (2.3)) is also dependent on the $\left(\Delta_{\text{resp}}^{14}-\Delta_{\text{meas}}^{14}\right)$ difference (figure 5).

#### (iv) Overall uncertainty of ΔffCO_2_ for typical ICOS stations

**Figure 7.**
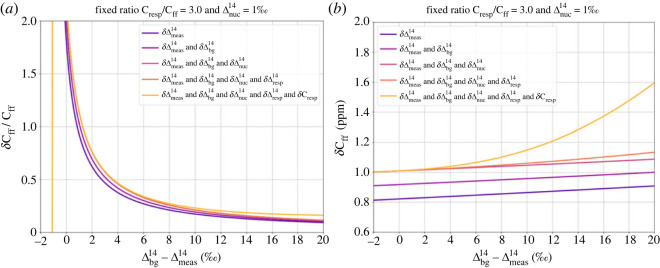
(*a*) Relative uncertainty of ΔffCO_2_ in relation to the observed difference of Δ^14^ between background and station $\left(\Delta_{\text{bg}}^{\text{14}}-\Delta_{\text{meas}}^{14}\right)$*.* (*b*) The absolute uncertainty in ppm. Note, the *x*-axis starts at −2 to account for masking effects.

**Figure 8.**
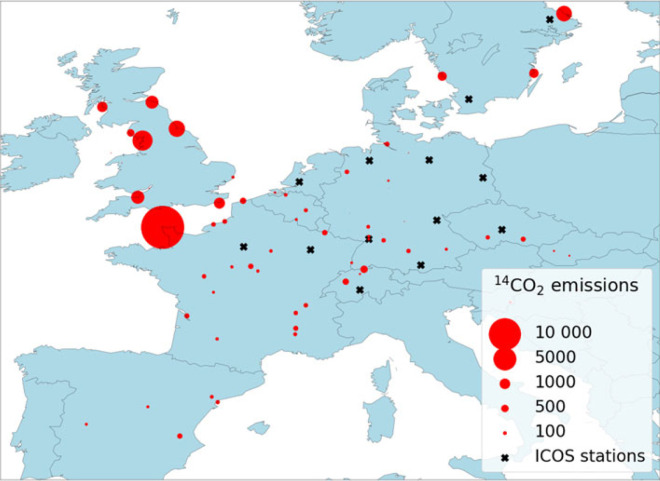
Nuclear ^14^CO_2_ emissions in Europe (annual means for 2018). The black crosses show the locations of the ICOS class-1 stations. See the text for a description of how this map was created.

The assumed concrete values and parameters that were used in the following overall error estimate of ΔffCO_2_ derived from Δ^14^CO_2_ measurements of two-week integrated and flask samples at typical ICOS stations and their assumed uncertainties are listed in table 1. Figure 7*a* shows the relative uncertainty of bias-corrected ΔffCO_2_ in relation to the difference of Δ^14^ between background and station $\left(\Delta_{\text{bg}}^{14}-\Delta_{\text{meas}}^{14}\right)$, while figure 7*b* shows the absolute uncertainty in ppm. The different colours show different combinations of individual uncertainty contributions. It is obvious that the largest contribution to the overall uncertainty of bias-corrected ΔffCO_2_ is due to the analytical error of the ^14^C measurements, $\Delta_{\text{meas}}^{14}$. When adding an uncertainty of the background reference $\Delta_{\text{bg}}^{14}$ of 1‰ and a typical nuclear contamination of $\Delta_{\text{nuc}}^{14}=(1\pm 1)\text{‰}$, the overall uncertainty of (bias-corrected) ΔffCO_2_ is larger than 50% at an observed $\left(\Delta_{\text{bg}}^{\text{14}}-\Delta_{\text{meas}}^{14}\right)$ difference smaller than 3.5‰. (Note that the uncertainty of C_meas_ and C_bg_ have not been added in figure 7 as both error contributions are negligible.) Considering the median $\left(\Delta_{\text{bg}}^{14}-\Delta_{\text{meas}}^{14}\right)$ value (figure 6*a*,*b*), this means that, when ignoring the uncertainty due to respiration masking (yellow curve in figure 7), almost half of the ^14^C-based ΔffCO_2_ estimates from integrated samples at ICOS stations have an uncertainty greater than 50%. Only at observed $\left(\Delta_{\text{bg}}^{14}-\Delta_{\text{meas}}^{14}\right)$ differences larger than 8‰ the uncertainty of the ΔffCO_2_ estimate becomes smaller than 25%. This illustrates the importance of precise Δ^14^CO_2_ measurements. If the Δ^14^CO_2_ measurement uncertainty can be reduced to 1‰, only about one-third of the integrated samples at ICOS stations would have an ΔffCO_2_ uncertainty greater than 50%.

**Figure 9.**
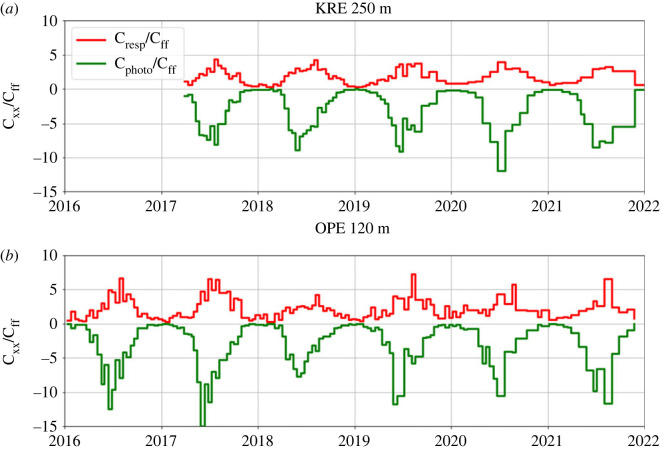
Simulated C_photo_/C_ff_ (green) and C_resp_/C_ff_ (red) ratios for the integrated samples collected at the two ICOS sites KRE (*a*) and OPE (*b*).

Figure 7*b* shows the absolute uncertainty of bias-corrected ΔffCO_2_ in ppm. The absolute error is only slightly increasing to 1.1 ppm (for an assumed 2‰ $\Delta_{\text{meas}}^{14}$ uncertainty) with larger differences between background reference and station $\left(\Delta_{\text{bg}}^{14}-\Delta_{\text{meas}}^{14}\right)$. The yellow curves in figure 7*a*,*b* show the uncertainty contribution from C_resp_, when assuming that the respiration CO_2_ (C_resp_) signal is three times higher than C_ff_. During summer, when the $\left(\Delta_{\text{bg}}^{14}-\Delta_{\text{meas}}^{14}\right)$ difference is small, the C_resp_/C_ff_ is often larger than 3 (figure 9); however, in connection with low $\left(\Delta_{\text{bg}}^{14}-\Delta_{\text{meas}}^{14}\right)$ differences the error contribution from respiration masking will still be small. Contrary, for situations with high fossil components, i.e. during winter, we expect a ratio of less than 3; therefore, we conclude that an uncertainty contribution of 0.5 ppm due to respiration masking correction, as shown in figure 7*b*, probably represents an upper limit. All-in-all, however, the potential uncertainty of ffCO_2_ masking by ^14^C-enriched soil respiration is non-negligible and potentially of similar magnitude as the uncertainty of the contamination from nuclear installations, if the respiration CO_2_ is significantly ^14^C-enriched compared with that of the ambient atmosphere.

## 4. Discussion and conclusion

The property of fossil emissions being free from ^14^C makes Δ^14^CO_2_ a unique and direct tracer for the fossil share in regional CO_2_ excess compared with a (clean air) background. However, this method, called here the regional isotope budget approach, also has its challenges and uncertainties. In this study, we revisited the assumptions made to address these various challenges and gave an overall uncertainty of the ^14^C-based ΔffCO_2_ estimates so that those could be used in atmospheric inversion modelling to verify or evaluate fossil fuel emissions. In the following, we will summarize and discuss our main findings.

The first and most important decision is the choice of the background site, as the Δ^14^CO_2_ difference between observation and background site directly determines the ΔffCO_2_ concentrations. In general, the background site should be chosen to suit the target area of the ffCO_2_ emission estimates. For example, if the emissions of a city are to be sampled, it might be convenient to measure the signals from the city downwind and to use a regional or local background station upwind of the city (e.g. [14]). In our study, we wanted to select a background site for a station network like ICOS that covers wide parts of the European continent. If the ΔffCO_2_ estimates derived with the regional isotope budget approach shall be used as a constraint in an inversion modelling framework to evaluate the fossil fuel emissions, the used background site has to be representative for all boundaries of the targeted domain. Otherwise, ΔffCO_2_ signals originating from outside the domain would be distributed within the domain. For the European example, STILT back-trajectories suggest that the entire European domain is dominated by westerly winds in about two-thirds of all situations. Therefore, we chose MHD, located on the west coast of Ireland, as our background station. To construct a background Δ^14^CO_2_ curve, we calculated a smooth fit through the integrated Δ^14^CO_2_ observations of MHD and estimated its construction uncertainty to be less than 1‰. However, the clean Atlantic air background from MHD might be less representative for potentially slightly polluted continental air masses with non-western origin, which are expected to occur in about one-third of all situations. The lack of representativeness of the MHD reference for these situations induces a bias and an additional uncertainty to the ΔffCO_2_ estimates. We used the global TM3 model [36] and switched off the emissions from the European target area to estimate this representativeness bias and additional uncertainty. The bias depends on the distance between the observation site and the boundaries of the target area, as it affects the dilution of the background signal from the boundaries to the observation site. This means that potential observation sites close to the, e.g. eastern boundary of the target domain, can show substantial biases for situations when they are affected by CO_2_ plumes from outside the target region. Therefore, the ΔffCO_2_ estimates from such sites should be corrected for those biases if the ΔffCO_2_ observations are to be used in inverse models to evaluate ffCO_2_ emissions. Concerning the current ICOS atmosphere stations, these plumes from outside the European domain are typically well mixed when they arrive at the observation site; here we expect mean biases of order 0.1 ppm. Therefore, we think that it is appropriate to use MHD as a single background site for the observation sites in Central Europe and for all wind conditions. Furthermore, we recommend to not exclude data under easterly wind conditions as this could lead to sampling biases if the ΔffCO_2_ estimates are used in an atmospheric transport inversion to optimize ffCO_2_ emissions. However, the variability in the bias induces a representativeness uncertainty of the MHD reference, which must be added quadratically to the MHD background fit uncertainty. This representativeness uncertainty depends also on the sample type. For Křešín (KRE), which is an eastern ICOS site in Central Europe and therefore expected to be most influenced by a false representation of the eastern boundary, the standard deviation of the bias amounts to 0.12 ppm for two-week integrated samples and 0.28 ppm for potential midday flasks; these could have a bias of up to 2 ppm. The overall background Δ^14^CO_2_ uncertainty (0.9‰ for two-week integrated samples and 1.0‰ for flasks), i.e. the combination of the MHD fit and representativeness uncertainties, together with the measurement uncertainty of the Δ^14^CO_2_ signals at the monitoring sites (*ca* 2‰), account for *ca* 0.9–1.0 ppm uncertainty of the ΔffCO_2_ estimates for typical signals observed at ICOS sites (table 2).

**Table 2.** Contributions to the overall ΔffCO_2_ uncertainty. The shown uncertainties are representative of a range of $\left(\Delta_{\text{bg}}^{14}-\Delta_{\text{meas}}^{14}\right)$ differences from −2 to 20‰, which covers most of the observed range in figure 6. The uncertainty contribution of a certain parameter is the increase in the ΔffCO_2_ uncertainty if the uncertainty of this parameter is (quadratically) added to the ΔffCO_2_ uncertainty, which was caused by the previous parameters (in the rows above this certain parameter).

parameter	contribution to overall ΔffCO_2_ uncertainty	parameter value ± uncertainty
$\Delta_{\text{meas}}^{14}$	(0.8–0.9) ppm	± 2‰
$\Delta_{\text{bg}}^{14}$	0.1 ppm	(3 ± 1)‰
$\Delta_{\text{nuc}}^{14}$	0.1 ppm [0.3 ppm]	(1 ± 1)‰ [(2 ± 2)‰]
$\Delta_{\text{resp}}^{14}$	(0–0.05) ppm	(25 ± 12)‰
${\text{C}}_{\text{resp}}/{\text{C}}_{\text{ff}}$	(0–0.5) ppm	3 ± 3
${\text{C}}_{\text{bg}}$	∼10^−4^ ppm	(410 ± 1) ppm
${\text{C}}_{\text{meas}}$	∼10^−9^ ppm	(420 ± 0.1) ppm

In Europe, with more than 170 in-operation reactors and two reprocessing plants [41], the nuclear contamination of the Δ^14^CO_2_ samples is a serious problem. The median nuclear contamination at the ICOS sites accounts for about 30% (for day-and-night integrated samples) and almost 15% (for flasks that are collected during midday integrated over 1 h) of the median Δ^14^CO_2_ depletion compared with the MHD reference. This would roughly result in a 25% and 15%, respectively, underestimation of the ΔffCO_2_ estimates if no correction is applied. Therefore, we strongly recommend correcting the ΔffCO_2_ estimates for nuclear contaminations, especially for sites with small signals or substantial influence from nuclear facilities. The uncertainty for the nuclear contaminations originates from the transport model uncertainty as well as the uncertainty of the nuclear ^14^CO_2_ emissions. The transport model uncertainty depends on the sample type and the distance to the nuclear facilities. On the one hand, the nuclear corrections might be more reliable for integrated samples than for flasks as it is less relevant when exactly the plume of a nuclear point source hits the observation site. On the other hand, integrated samples include also night-time situations when transport models show typically poorer performance. The uncertainty of the nuclear emissions is mainly based on the strong temporal variability of the emissions [30,42], which we could not consider as we only had access to the officially reported annual mean ^14^CO_2_ emissions. Therefore, we assumed an uncertainty of 100% for the nuclear corrections. This increases the overall uncertainty of the nuclear corrected ΔffCO_2_ estimates by about 0.1 ppm for a typical nuclear contamination of 1‰. Overall, and particularly for flask sampling, we suggest calculating near-real time (or even better forecast) back-trajectories or footprints so that sampling during situations with potentially large nuclear influences can be avoided.

Soil respiration CO_2_ is expected to be a few tens of ‰ enriched in Δ^14^CO_2_ compared with atmospheric CO_2_, since it was initially fixed by plants during higher atmospheric Δ^14^CO_2_ levels after the atmospheric nuclear bomb testing. Therefore, also respiration CO_2_ may lead to masking part of the ΔffCO_2_ signal, depending on the Δ^14^CO_2_ difference between respired and atmospheric CO_2_ and the relative amount of respired CO_2_. For typical winter C_resp_/C_ff_ ratios of 1 (figure 9), the ΔffCO_2_ masking is less than 5% if the respiration Δ^14^CO_2_ signature is enriched by less than 40‰ compared with the ambient Δ^14^CO_2_. However, this masking effect can easily increase for a larger relative share of respiration CO_2_, e.g. during summer, and with higher soil respiration Δ^14^CO_2_ signatures. For a typical summer C_resp_/C_ff_ ratio of 6 at ICOS sites, the ΔffCO_2_ masking reaches already almost 20% for a 40‰ Δ^14^CO_2_ enrichment in the respired CO_2_. Therefore, the correction for soil respiration is especially important for sites with low ffCO_2_ signals and strong biosphere influence, which might be the case for many ICOS sites. The respiration CO_2_ component in the ambient CO_2_ concentration is hard to estimate, particularly during the day when photosynthesis is active. In these situations, a vegetation model in combination with an atmospheric transport model is needed. Therefore, we assume an uncertainty of 100% for the respiration CO_2_. Moreover, we estimated the uncertainty of the respiration Δ^14^CO_2_ signature to 50% of the Δ^14^CO_2_ enrichment compared with the ambient Δ^14^CO_2_. With those uncertainty assumptions, we expect the contribution of the uncertainty of the biosphere correction to the overall ΔffCO_2_ uncertainty to be smaller than 0.5 ppm for typical ICOS sites.

In this study, we also investigated the effect, if the Δ^14^CO_2_ signature of the photosynthetically absorbed CO_2_ is approximated with the ambient air Δ^14^CO_2_ at the measurement site (see equation (2.3)) or, alternatively, with the background Δ^14^CO_2_ observations (see equation (2.4)). It turns out that the relative bias in the ΔffCO_2_ estimates caused by these two different approximations is typically restricted to a few per cent only. The choice between equation (2.3) or equation (2.4) to calculate the ΔffCO_2_ concentrations may also depend on the availability of the required measurements. For example, equation (2.4) requires CO_2_ concentration data at the observation site. However, in some cases, the CO_2_ concentration at the observation site is not known, e.g. if the ΔffCO_2_ is based on Δ^14^C from plant material. For such cases, equation (2.3) might be more useful, as its major term depends on the background CO_2_ concentration C_bg_ instead of C_meas_. However, C_meas_ is also needed in the nuclear correction term (second term of equation (2.3)). We, therefore, investigated the bias if C_meas_ is replaced by C_bg_ in equation (2.3). Obviously, it depends on the CO_2_ concentration difference between C_meas_ and C_bg_ and the magnitude of the nuclear contamination. Even if the nuclear contamination is of the order of the Δ^14^ difference between measurement and background site, this bias will still be below 5% for CO_2_ differences (C_meas _− C_bg_) of up to 40 ppm. Thus, this effect is much smaller compared with the bias introduced by neglecting the nuclear correction itself.

Overall, this study shows that the largest single uncertainty contribution to ΔffCO_2_ estimates still comes from the limited precision in Δ^14^CO_2_ analyses if the potential biases due to nuclear contamination or biosphere respiration are corrected for. Even a Δ^14^CO_2_ measurement uncertainty of 1‰ would still make up a substantial contribution to the overall ΔffCO_2_ uncertainty for typical $\left(\Delta_{\text{bg}}^{\text{14}}-\Delta_{\text{meas}}^{14}\right)$ gradients at ICOS sites and assumed nuclear $\Delta_{\text{nuc}}^{14}$ contributions of 1‰. This means that about every second (or third) integrated sample from the ICOS network would yield a ΔffCO_2_ uncertainty larger than 50% for an assumed 2‰ (or 1‰) Δ^14^CO_2_ measurement uncertainty. These large relative ΔffCO_2_ uncertainties can be explained by the small $\left(\Delta_{\text{bg}}^{\text{14}}-\Delta_{\text{meas}}^{14}\right)$ gradients at the remote ICOS sites. Therefore, the regional isotope budget approach is best suited in polluted areas with large $\Delta_{\text{meas}}^{14}$ depletions due to fossil CO_2_ emissions. In such cases, the regional isotope budget approach is also relatively less sensitive to possible representativeness biases in $\Delta_{\text{bg}}^{14}$. If the transport-driven variability in the ΔffCO_2_ estimates can be reproduced by an atmospheric transport model, the ΔffCO_2_ observations can be used in inversion frameworks to estimate ffCO_2_ emissions. Graven *et al*. [18] used ^14^C-based ΔffCO_2_ observations from nine urban and non-urban sites in California to investigate the ffCO_2_ surface fluxes with a Bayesian inversion framework based on Fischer *et al*. [43]. They could show that their estimates for the total in-state emissions are consistent with bottom-up estimates. Thus, it would also be interesting to investigate if the ΔffCO_2_ observations from the ICOS sites can be used to estimate the ffCO_2_ emissions in Europe. Indeed, the signal-to-noise constraints that limit the ΔffCO_2_ detection at remote sites might not limit the inverse problem in the same way. Thus, the large and growing number of samples from distributed ICOS sites with small but persistent ΔffCO_2_ signals may provide meaningful adjustments of the ffCO_2_ surface fluxes in Central Europe. An alternative approach to investigate ffCO_2_ emissions is the dual-tracer inversion, which uses the CO_2_ and Δ^14^CO_2_ observations directly. This approach has already successfully been applied in the US by Basu *et al*. [10]. The authors used the CO_2_ and Δ^14^CO_2_ observations from the NOAA station network in North America and showed that the estimated US national total ffCO_2_ emissions for 2010 are significantly larger than reported by bottom-up inventories. Undoubtedly, this dual-tracer inversion approach should also be implemented in Europe to take advantage of the ICOS observation network. Ideally, the results of the ffCO_2_ inversion and the dual-tracer inversion would then converge within their individual uncertainty margins.

Certainly, a big challenge in Europe is the large number of nuclear power plants [29], which affect both, the regional isotope budget approach, and the dual-tracer inversion approach. Thus, there is an urgent need for temporally highly resolved (i.e. at least monthly or weekly) ^14^CO_2_ emissions data from nuclear facilities in Europe, so that reliable and un-biased ffCO_2_ emission estimates can be deduced. In other parts of the world with a much lower spatial density of nuclear installations this ‘nuclear problem’ seems to be less prominent than in Europe. However, we showed that the biosphere respiration could lead to similar biases as the nuclear contaminations if not properly taken into account. In other rural regions of the world the respiration $\Delta_{\text{resp}}^{14}$ might be different, depending on climate and vegetation types. This must be considered, so that ΔffCO_2_ concentrations can be estimated bias-free. But also, the dual-tracer inversion requires a reliable *a priori* representation of the $\Delta_{\text{resp}}^{14}$ signature.

Finally, we showed that a marine site located at the western edge of the European target region is an appropriate background site for calculating ΔffCO_2_ estimates for sites in Central Europe. Similarly, Graven *et al*. [18] used measurements from three coastal sites in California to construct a Δ^14^CO_2_ background for observation sites in California. Zhou *et al*. [17] defined the Qixianling Mountain, which is located on the Hainan Island in the south of China as a background to estimate ΔffCO_2_ concentrations for 15 Chinese cities. To conclude, if the Δ^14^C observations of a single site are used to estimate ΔffCO_2_ concentrations for an observation network, a potential background representativeness bias and uncertainty should be investigated by either comparing the $\Delta_{\text{bg}}^{14}$ values with observations from other potential background sites or estimating the representativeness bias via model simulations as in the present study.

## Data Availability

This article has no additional data. Radiocarbon observations from the ICOS sites can be downloaded from the ICOS Carbon Portal (https://www.icos-cp.eu/, last access: 29 August 2023).

## Appendix A

**A.1. Nuclear contaminations and their uncertainties**

We used the ^14^CO_2_ emissions from the European Commission RAdioactive Discharges Database (RADD) where the member states of the European Union report their annual nuclear emissions [44]. For some facilities, only the total ^14^C emission but not the ^14^CO_2_ emission is reported. In these cases, we assume that pressurized water reactors (PWRs) emit 28% of the ^14^C as ^14^CO_2_ and that all other reactor types emit all ^14^C as ^14^CO_2_ according to Zazzeri *et al*. [45] and Graven & Gruber [29]. Since the Belgian facilities are missing in the RADD database, we used the energy consumption data from the International Atomic Energy Agency-Power Reactor Information System (IAEA-PRIS, available at: https://pris.iaea.org/PRIS/CountryStatistics/CountryDetails.aspx?current=BE, last access: 5 October 2022) and multiplying a factor of 0.24 TBq/a per GWa for PWRs and 0.51 TBq/a per GWa for boiling water reactors to get the annual ^14^C emissions (as [45]). For the nuclear facilities in Switzerland the ^14^CO_2_ emissions were taken from the annual Radiological Protection Reports (available at: https://www.ensi.ch/en/documents/document-category/strahlenschutzberichte/, last access: 5 October 2022) authored by the Swiss Federal Nuclear Safety Inspectorate (ENSI). This nuclear ^14^CO_2_ emission map (figure 8) has been implemented at the ICOS CP.

To calculate the Δ^14^CO_2_ contaminations from nuclear facilities at the observation site $\left(\Delta_{\text{nuc}}^{14}\right)$, we transport the ^14^CO_2_ emissions with the STILT model [26]. In STILT, the surface source influence *f* with units (µmol/mol_air_)/(µmol/m^2^s) describes the sensitivity to emissions from surface grid cells (*x_i_*, *y_j_*) in the footprint of the station. As the nuclear ^14^CO_2_ emissions *Q* are given in units Bq/(m^2^s), the mapping of those emissions with the surface source influence *f* yields a nuclear activity *A*_S_ = (*Q* o *f*) in units Bq/mol_air_ (‘o’ indicates the element-wise multiplication of each surface influence grid cell (*x_i_*, *y_j_*) with its corresponding flux *Q*(*x_i_*, *y_j_*) and the subsequent sum over all grid cells). Equation (A 1) gives the definition of the Δ-notation according to Stuiver & Pollach [24] and equation (A 2) the $\Delta_{\text{nuc}}^{14}$:

A\,1 $$\Delta^{14}{\text{CO}}_{\text{2}}^{\;}=\left(\frac{A_{\text{SN}}}{A_{\text{abs}}}-1\right)\cdot 1000\text{‰}\approx \;\left(\frac{0.968\cdot A_{\text{S}}}{A_{\text{abs}}}-1\right)\cdot 1000\text{‰}$$

and

A\,2 $$\Delta_{\text{nuc}}^{14}=\frac{0.968\cdot (Q\circ f)}{{\text{C}}_{\text{meas}}\cdot M_{\text{C}}\cdot A_{\text{abs}}}\cdot 1000\text{‰}.$$

Here, we assume a constant atmospheric δ^13^C–CO_2_ value of −9‰ and use the standard activity *A*_abs_ of 0.226 Bq/gC (‘gC’ means gram carbon). To get the sample activity in the same units as the standard activity, we have to divide the sample activity by the product of the CO_2_ concentration at the measurement site $C_{\text{meas}}$ and the molar mass of carbon $M_{C}$. Since the RADD nuclear emissions are reported only on an annual basis, we assume constant emissions throughout the year. This procedure of mapping the RADD ^14^CO_2_ emissions with modelled surface source influences has been implemented in the radiocarbon Jupyter notebook offered by the ICOS Carbon Portal. The surface source influence fields are available for all ICOS sites on a three-hourly resolution and were generated with 0.25° resolution meteorological data from the European Center for Medium-Range Weather Forecasts (ECMWF). Since the nuclear emissions are released from stacks with a height of typically above 100 m, using the standard STILT approach that can transport only surface emissions, may overestimate ^14^CO_2_ activity concentrations during stable (night-time) conditions if the nuclear installations are located closer than *ca* 50 km from the measurement site. For these stations a more sophisticated approach introduced by Maier *et al*. [27] should be applied. In the current study we did, however, only use the standard surface influence approach and the distribution of $\Delta_{\text{nuc}}^{14}$ for integrated samples shown in figure 6*b* may, thus, be slightly biased high for some of the stations (i.e. NOR).

The uncertainty of the transport model is mainly due to the uncertainty of the meteorological input fields and their resolution as well as on the correct representation of the effective emission heights. Kuderer *et al*. [30] showed with the Hybrid Single-Particle Lagrangian Integrated Trajectory (HYSPLIT) model that the mean nuclear contamination from nearby power plants can be more than doubled if the horizontal resolution of the meteorological fields is increased from 0.5° to 2.5°. Therefore, we used high-resolution meteorological fields from ECMWF. Considering these various and difficult-to-quantify sources of uncertainty to calculate $\Delta_{\text{nuc}}^{14}$, we chose a simple but justified approach and applied an uncertainty of 100% for all sample types. This allows on the one hand to cover situations with the dominant nuclear ^14^CO_2_ emissions during the two-week period being twice as high as expected (i.e. the annual average). On the other hand, it considers situations when the model erroneously predicts nuclear influences at the observation site.

**A.2. Construction of the MHD $\Delta_{\text{bg}}^{14}$ background curve**

In this study, we use the two-week integrated Δ^14^CO_2_ data from the maritime site MHD (53.33° N, 9.90° W, 5 m a.s.l.) located at the west coast of Ireland to calculate marine background reference curves for all ICOS stations. We applied the curve fitting algorithm from the NOAA to the two-week integrated Δ^14^CO_2_ measurements. A description of the fitting routine can be found at: https://gml.noaa.gov/ccgg/mbl/crvfit/crvfit.html and a Python script is freely available at: https://gml.noaa.gov/aftp/user/thoning/ccgcrv/ (last access: 11 March 2022). We used the following three components of the NOAA curve fitting routine: (i) a polynomial function with three terms (i.e. a quadratic function) to account for the long-term Δ^14^CO_2_ decline, (ii) two harmonics to simulate the annual Δ^14^CO_2_ cycle and (iii) a low-pass filter to the residuals with a long-term cut-off value of 5 years to consider interannual variations. This NOAA fitting routine also provides variances $\sigma_{\text{func}}^{2}$ and $\sigma_{\text{filt}}^{2}$ for the function, which is set up by the polynomial terms and the harmonics, and for the low-pass filtering of the residuals, respectively. The total variance $\sigma_{\text{fit}}^{2}$ of the NOAA fit is then given by the combination of both variances (see https://gml.noaa.gov/ccgg/mbl/crvfit/crvfit.html, equation (A 3)):

A\,3 $$\sigma_{\text{fit}}^{2}=\sigma_{\text{func}}^{2}+\sigma_{\text{filt}}^{2}.$$

The NOAA fitting routine was originally developed for CO_2_ concentrations, which can be measured very accurately with uncertainties of typically below 0.1 ppm. So, the ratio between measurement uncertainty and a typical seasonal variation of 20 ppm is only 5‰. That is why the measurement uncertainties of the individual observations are not considered by the NOAA fit. However, in the case of Δ^14^CO_2_ the measurement uncertainty is *ca* 2‰, which is 40% of a typical 5‰ peak-to-peak seasonal Δ^14^CO_2_ variation at background sites. Therefore, we also performed a Monte Carlo approach, to take into account the measurement uncertainties of the individual two-week integrated Δ^14^CO_2_ samples. We draw for each Δ^14^CO_2_ sample a random number from a Gaussian distribution with zero mean and the one-sigma standard deviation given by the respective measurement uncertainty. The initial Δ^14^CO_2_ measurements were then shifted by those random numbers. Afterwards, we calculated the NOAA fitting curve for this configuration. After repeating this procedure 1000 times, we could calculate the standard deviation over all 1000 background curves to get an additional estimate of the average background curve uncertainty.

The NOAA fitting routine yields a standard deviation for $\Delta_{\text{bg}}^{14}$ of 0.86‰ for the points relative to the background curve, while the standard deviation over the 1000 Monte Carlo realizations was on average only 0.43‰, i.e. half the standard deviation calculated with the NOAA fitting algorithm. This small standard deviation of the Monte Carlo realizations shows that the chosen parameters (i.e. the long-term cut-off value of 5 years) leads to a background curve, which is robust to variations within the one-sigma range of the individual Δ^14^CO_2_ measurements. If we vary the individual Δ^14^CO_2_ measurements within their two-sigma uncertainty range, we get a standard deviation of 0.86‰ over the 1000 Monte Carlo realizations, i.e. the same value as the standard deviation computed with the NOAA algorithm. We also investigated the influence of a different number of polynomials (two or four polynomials instead of three), harmonics (three or four harmonics instead of two) and years for the long-term cut-off value (1 or 3 years instead of 5). These parameter-varied background curves lie all well within the 0.86‰ uncertainty band of the MHD background curve. On average, the deviations are even less than 0.1‰. That is why we will use in the following the 0.86‰ standard deviation as an estimate for the uncertainty of the MHD background Δ^14^CO_2_ curve.

**A.3. Modelled C_resp_/C_ff_ and C_photo_/C_ff_ ratios for two ICOS sites**

Figure 9 shows the modelled C_photo_/C_ff_ and C_resp_/C_ff_ ratios for the integrated samples collected at the two ICOS sites Křešín (KRE) in the Czech Republic and Observatoire Pérenne de l'Environnement (OPE) in France between 2016 and 2021. We used VPRM-STILT to simulate the biospheric CO_2_ signals and the Emissions Database for Global Atmospheric Research (EDGAR, [46]) and STILT to calculate the C_ff_ contributions. The model results illustrate the expected range of the ratios at typical ICOS sites in Central Europe as well as their seasonal cycle with lower (absolute) ratios in winter compared with summer.
